# What are the health impacts of nicotine and tobacco products on young people?

**DOI:** 10.1038/s41432-023-00945-w

**Published:** 2023-10-19

**Authors:** Chris Deery

**Affiliations:** School of Clinical Dentistry, University of Sheffield, Sheffield, UK

**Keywords:** Oral diseases, Tobacco cessation in dentistry

## Abstract

**Design:**

Narrative review.

**Review question:**

What are the implications of new nicotine and tobacco products on adolescent health?

**Products:**

E-cigarettes and oral nicotine products such as pouches, lozenges, tablets, gum, and gummies.

**Results:**

These products can be addictive and can cause respiratory, cardiovascular and oral potential health problems. They also have effects on brain development. Products are attractive to adolescents, with more than 1:10 American adolescents uses them.

**Conclusions:**

The use of nicotine and tobacco products by adolescents puts their health at risk and may, in some instances, lead to nicotine addiction. Those providing healthcare for adolescents have an opportunity to provide advice and signpost people to resources to help them stop using such products. There is also a need for legislation to restrict the sale of e-cigarettes and tobacco products.

## A Commentary on

**Heinly A, Walley S**.

The nicotine and tobacco epidemic among adolescents: new products are addicting our youth. *Curr Opin Pediatr* 2023; **35:** 513–521.

**GRADE Rating:** 

## Commentary

This narrative review discusses the use of nicotine and tobacco products particularly by adolescents and young people. It discusses the position, including the legislative position in the United States of America (USA). However, this does not make it any less relevant to readers elsewhere in the World. Electronic cigarettes (e-cigarettes) were first available in the mid-2000s^1^. Recently other oral nicotine products such as pouches, lozenges, tablets, gum, and gummies, have become available. E-cigarettes have also developed through a number of generations from products that looked like cigarettes to products to the more recent disposable products. These latter products are particularly attractive to adolescent users^2^. USA data reports 11.3% of all school students using tobacco products in some form^3^.

Products are frequently flavoured making them appealing to adolescents, common flavours include fruit, candy, dessert or other sweet tastes.

The use of e-cigarettes and nicotine products is linked to a number of health issues. Levels of nicotine absorbed varies with products and usage. Nicotine is addictive, use of nicotine products is characterised by strong cravings, physiological withdrawal symptoms, with increasing tolerance that results in increased usage^4^. Levels of nicotine absorbed varies with products and usage. Exposure to nicotine is not the only health issue. E-liquid can also contain potentially toxic substances and carcinogens^5^. Other potential health concerns include:

- Nicotine is damaging to the developing brain^6^
- There is an association between e-cigarette use and subsequent use of traditional cigarettes smoking, marijuana, alcohol and amphetamines^1,4^
- Lung damage, shortness of breath and asthma^7,8^
- Possible cardiovascular health effects^9^

Regarding oral health, e-cigarettes are linked to oral dryness, burning, irritation, bad taste halitosis, pain/discomfort, mucosal lesions, black tongue and burns^10^. There is also an increased incidence of periodontal and gingivitis^10^.

The American Academy of Pediatrics Ask-Councel-Treat model is presented as a way for health professional to approach supporting adolescents stop the use of tobacco products^11^. This starts by screening patients by asking them if they use tobacco or vaping products. This is followed by the provision of information about the potential effects on health of these products. Other benefits including financial and independence are also discussed before asking if the person wants to stop. The person is then signposted to where they will get help is stop or “quit”.

Tobacco and nicotine product use is linked to significant detrimental effects on health particularly the health of adolescents.

Practice points

- Ask all adolescent patients about their tobacco and nicotine product use.
- Provide them with information about the potential effects on health of tobacco and nicotine products.
- Know what resources are available to sign-post patients who indicate they wish to stop.
